# Prevalence and Antimicrobial Susceptibility of *Salmonella* in Retail Meat Collected from Different Markets in Sichuan, China

**DOI:** 10.3390/pathogens14030222

**Published:** 2025-02-25

**Authors:** Hang Zeng, Donghai Yang, Nanxi Huang, Yonglin Li, Jiazhen Chen, Zhongjia Yu, Jie Tang, Zhenju Jiang

**Affiliations:** 1 School of Food and Bioengineering, Xihua University, Chengdu 610039, China; 2 Food Microbiology Key Laboratory of Sichuan Province, Xihua University, Chengdu 610039, China; 3 School of Animal Science and Technology, Foshan University, Foshan 528225, China

**Keywords:** *Salmonella*, retail meat, prevalence, serovar, antimicrobial resistance

## Abstract

*Salmonella* is one of the most significant zoonotic and foodborne pathogens, and it is the leading cause of bacterial diarrhea. In this study, 156 retail meat samples were collected from three supermarkets and one local wet market in Sichuan, China, including 96 chicken samples and 60 pork samples. The prevalence of *Salmonella* in these samples was analyzed, and 91 samples (58.33%) tested positive, with 60 (62.5%) positive chicken samples and 31 (51.67%) positive pork samples. From these positive samples, 190 *Salmonella* isolates were confirmed by double PCR. Subsequent serotyping identified nine serovars, with the predominant ones being *S.* London (58.94%), *S.* Typhimurium (12.58%), and *S.* Enteritidis (10.60%). Antibiotic susceptibility test revealed that 168 isolates (88.42%) were resistant to at least one antibiotic, and 150 isolates (78.95%) were resistant to three or more antibiotics. The highest resistance rates were observed for ampicillin (83.16%), followed by tetracycline (76.31%) and trimethoprim/sulfamethoxazole (67.37%). In the disinfectant susceptibility test, *Salmonella* isolates exhibited higher resistance rates to benzalkonium bromide (100%) and benzalkonium chloride (97.37%), while showing a lower resistance rate to potassium monopersulfate triple salt (33.6%). These findings highlight the high prevalence of *Salmonella* and its significant resistance to antibiotics and disinfectants, indicating that effective measures must be implemented to ensure the microbiological safety of retail meat.

## 1. Introduction

The genus *Salmonella* belongs to the family Enterobacteriaceae and consists of two species, *S. enterica* and *S. bongori*. So far, a total of 2659 *Salmonella* serovars have been identified, of which 2637 serovars belong to *S. enterica* [1]. The species *S. enterica* consists of typhoidal and non-typhoidal *Salmonella* (NTS) and is responsible for the majority of human and animal *Salmonella* infections. NTS is one of the most frequently reported foodborne pathogens that cause diarrhea around the world, which is responsible for 180 million diarrhea cases yearly [2]. Livestock and poultry meat are the major *Salmonella* sources of human infections, and meat and meat products can be contaminated by *Salmonella* from farm to fork. In China, *Salmonella* accounts for around 70–80% bacterial foodborne disease outbreaks and is one of the top two bacterial pathogens causing diarrhea [3,4,5]. In addition, there is a significant consumption of pork and chicken meat in China. Sichuan Province stands as a prominent producer for both pork and chicken. Therefore, conducting research on *Salmonella* contamination in these meats will assist in identifying potential risks.

Recent years, the emergence and dissemination of multi-drug-resistant (MDR) *Salmonella* strains in animals and humans has become one of the top global public health threats. Especially, a multi-drug-resistant *Salmonella* Infantis (*S.* Infantis) strain that carries a pESI-like mega-plasmid, displaying escalating global prevalence [6]. This strain exhibits enhanced fitness, antimicrobial resistance (e.g., resistant to third-generation cephalosporins and quinolones), and other concerning traits (e.g., disinfectant resistance), posing a significant threat to human health [6].

In meat production, using disinfectants like quaternary ammonium or potassium persulfate is an effective measure to prevent microbial contamination including *Salmonella*. However, the improper use or overuse of disinfectants may lead to *Salmonella* developing resistance to disinfectants, resulting in a decrease in the disinfection efficiency and frequent cross-contamination in meat production [7,8,9,10]. Furthermore, studies have shown that the antibiotic susceptibility of *Salmonella* isolates could be changed under certain disinfectant stress, i.e., co-resistance of disinfectant-resistant bacteria to antibiotics [11,12]. In this way, eliminating *Salmonella* especially MDR *Salmonella* is becoming more and more challenging, posing great threat to public health.

To date, there have been no reports of the emerging *S.* Infantis strain in China; instead, *S.* Indiana has been frequently reported [13]. Therefore, in order to ascertain if the emerging *S.* Infantis strain is prevalent in Sichuan Province and to investigate the prevalent serovars of *Salmonella* within the region, in this study, we conducted an investigation into *Salmonella* contamination in pork and chicken meat sold at a local wet market and different supermarkets in Chengdu, Sichuan Province. Antibiotic susceptibility tests were performed on the isolated strains, and the minimum inhibitory concentration (MIC) for disinfectants were determined, with the aim of providing essential data that can serve as a basis for pig and poultry farming as well as production practices.

## 2. Materials and Methods

### 2.1. Sample Collection

From March to April 2023, a total of 156 samples including chicken (*n* = 96) and pork (*n* = 60) were collected from three supermarkets and a local wet market in Pidu district of Chengdu, Sichuan Province. Each sample was weighed, labeled, and placed into separate sterile bags and then immediately transported to the laboratory at low temperatures and processed within 4 h. Pork samples were pre-treated in accordance with the National Food Safety Standard GB 4789.4-2016 [14]. Chicken samples were pre-treated following the methods described by Hou et al. [15]. The details of samples are shown in Table 1.

### 2.2. Salmonella Isolation and Identification

The isolation and identification of *Salmonella* were carried out according to GB 4789.4-2016 [14]. Briefly, pre-treated samples were transferred to tetrathionate broth (TTB) and selenite cysteine (SC) broth (Hi-Tech Industrial Park Hope Bio-Technology, Qingdao, China) and incubated at 42 °C and 37 °C for 18–24 h, respectively. After enrichment, TTB or SC broth was picked and streaked on xylose lysine desoxycholate (XLD) agar (Hi-Tech Industrial Park Hope Bio-Technology, Qingdao, China), then incubated at 37 °C for 18 h. One or two colonies that appeared on the XLD agar and had suspicious appearance were picked from the XLD agar for further identification by double PCR assay, which would simultaneously amplify two specific genes (*invA* and *hut*) of *Salmonella* in a single PCR reaction. Briefly, synthesized primers amplifying *invA* gene (invA-F: 5′-GTGAAATTATCGCCACGTTCGGGCAA-3′, invA-R: 5′-TCATCGCACCGTCAAAGGAACC-3′) and *hut* gene (hut-F: 5′-ACTGGCGTTATCCCTTTCTCTGCTG-3′, hut-R: 5′-ATGTTGTCCTGCCCCTGGTAAGAGA-3′) were added into the PCR reaction mixture; PCR amplification (Biometra, Gottingen, Germany) was performed under the following conditions: initial denaturation at 94 °C for 5 min; 40 cycles of denaturation at 94 °C for 40 s, annealing at 60 °C for 40 s, and extension at 72 °C for 50 s; followed by a final extension at 72 °C for 5 min [16,17]. Final PCR products were visualized in 1.5% agarose gel, and two bands of 284 bp and 495 bp would be observed from the positive *Salmonella* strains.

In addition, the O and H antigens of *Salmonella* were serotyped by slide agglutination test using commercially available antisera following the manufacturer’s instructions (Tianrun Bio-Pharmaceutical, Ningbo, China).

### 2.3. Antimicrobial Susceptibility Test

After identifying *Salmonella* isolates, the susceptibility of all *Salmonella* isolates to a panel of 10 antibiotics was determined using the standard Kirby–Bauer disk diffusion method, as recommended by the Clinical and Laboratory Standards Institute (CLSI 2022) [18]. The doses and names of the antibiotics were as follows: ampicillin (AMP, 10 µg), cefazolin (CZ, 30 µg), ampicillin/sulbactam (AAM, 10/10 µg), amoxicillin/clavulanic acid (AMC, 20/10 µg), ciprofloxacin (CIP, 5 µg), amikacin (AK, 30 μg), gentamycin (GEN, 10 µg), tobramycin (NN, 10 µg), tetracycline (TET, 30 µg), and trimethoprim/sulfamethoxazole (SXT, 1.25/23.75 µg) (Hangzhou Microbiology Reagent, Hangzhou, China). As required by CLSI, *Escherichia coli* ATCC 25922 served as the control strain in the experiment. The results were considered valid only when the diameter of the inhibition zone fell within the acceptable range as delineated by the CLSI guidelines.

### 2.4. Determination of Minimum Inhibitory Concentration (MIC) of Disinfectants

The susceptibility of *Salmonella* isolates to three disinfectant agents was assessed using the broth micro-dilution method. The disinfectants used in this study included benzalkonium chloride (BC), benzalkonium bromide (BAB), and potassium monopersulfate triple salt (PMTS) (Macklin Biochemical Technology, Shanghai, China).

Before inoculation, the bacterial suspension was adjusted to 0.5 McFarland standard with sterile saline, and the suspension was diluted 100-fold with Mueller–Hinton broth (MHB; Hangzhou Microbial Reagent, Hangzhou, China). The prepared bacterial suspension was inoculated into a 96-well plate (final volume of 200 µL) with the completed dilution of the disinfectant solution and then incubated at 37 °C for 24 h. The MIC was determined as the lowest concentration of the disinfectant at which no visible bacterial growth was detected in the 96-well plate. *E. coli* ATCC 25922 was used as a quality control (QC) strain for disinfectant susceptibility test. The MIC values for the isolated *Salmonella* strains were compared to those of the QC strain. If the MIC values of the isolated strain was higher than the MIC value of the QC strain, it was classified as disinfectant-resistant strain [7].

## 3. Results

### 3.1. Prevalence and Identification of Salmonella Isolates

In this study, a total of 156 samples were subjected to isolation and identification, yielding 91 positive results. Among the positive samples, 60 were from chicken and 31 were from pork. These findings led to an overall contamination rate of 58.33% (91/156), with specific rates of 69.77% (60/96) for chicken-derived *Salmonella* and 51.67% (31/60) for pork-derived *Salmonella*. Among the four sampling sites, supermarket A and supermarket C had the highest contamination rate of 61.54% (24/39). In contrast, the contamination rates of the local wet market and supermarket B were lower, at 58.97% (23/39) and 51.28% (20/39), respectively.

From these 91 positive samples, 190 isolates were positive for *Salmonella* and confirmed by double PCR. For further identification, serotyping was performed, and the results are shown in Table 2. Overall, 151 *Salmonella* isolates were identified into 6 *Salmonella* serogroups representing 9 different *Salmonella* serovars, and the remaining 39 strains could not be typed. The most prominent *Salmonella* serogroups were E1 (102, 67.55%), B (23, 12.04%), and D1 (16, 10.60%). The main serovars were *S.* London (89, 58.94%), *S.* Typhimurium (12.58%), and *S.* Enteritidis (16, 10.60%), while *S.* Infantis was not detected in this study.

### 3.2. Antibiotic Resistance in Salmonella Isolates

**Figure 1:**
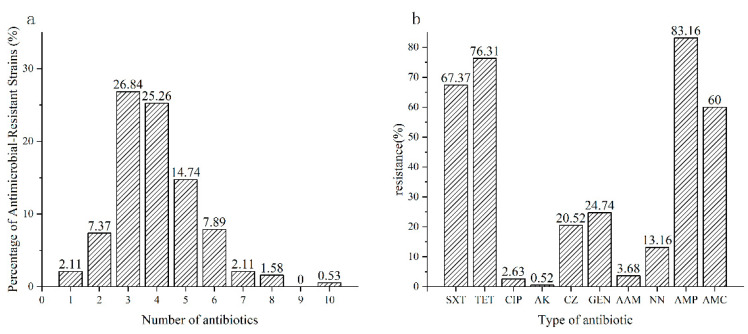
Antibiotic resistance of *Salmonella* isolates. (**a**) Antibiotic resistance rate of *Salmonella* isolates. (**b**) Distribution of antibiotic resistance in *Salmonella* isolates.

Antibiotic susceptibility was assessed after identifying the *Salmonella* isolates (Supplementary Materials Table S2). In 190 *Salmonella* strains, 168 (88.42%) isolates exhibited resistance to at least one antibiotic, and 150 (78.95%) isolates showed resistance to three or more antibiotics. As shown in Figure 1a, among the 168 resistant strains, 4 strains were resistant to only one antibiotic (2.10%), 14 strains were resistant to two antibiotics (7.37%); 51 strains were resistant to three antibiotics (26.84%); 48 strains were resistant to four antibiotics (25.26%); 28 strains were resistant to five antibiotics (14.74%); 15 strains were resistant to six antibiotics (7.89%); 4 strains were resistant to seven antibiotics (2.11%); 3 strains were resistant to eight antibiotics (1.58%); and 1 strain was resistant to ten antibiotics (0.53%). In terms of specific antibiotics, a higher prevalence of resistance to AMP (83.16%), TET (76.31%), SXT (67.37%), and AMC (60.00%) was observed. In comparison, resistance to GEN (24.71%), CZ (20.52%), and NN (13.16%) was observed less frequently. Most strains were sensitive to AAM (3.68%), CIP (2.63%), and AK (0.52%). Notably, only five isolates in this study were found to be resistant to ciprofloxacin (CIP); three of these were identified as *S.* Indiana, and the remaining one was *S.* Kentucky.

The antimicrobial susceptibility results at the primary serovars level are shown in Table 3. All strains of *S.* Muenster exhibited resistance to SXT, TET, CZ, GEN, and AMP. Additionally, 94.73% of *S.* Typhimurium displayed resistance to TET, AMP, and AMC. Among the five primary serotypes, *S.* Typhimurium was the most resistant serovars, with three strains exhibiting resistance to eight different antibiotics. This was succeeded by *S.* Muenster, which had one strain resistant to seven antibiotics.

Moreover, the antimicrobial resistance profile of *Salmonella* isolates is displayed in Table 4. In total, we observed 33 profiles with different combinations of antibiotics, and it was dominated by SXT-TET-AMP-AMC (34/168), followed by SXT-TET-AMP (25/168), TET-AMP-AMC (14/168), and SXT-TET-GEN-AMP-AMC (14/168).

### 3.3. Disinfectant Resistance of Salmonella Isolates

In addition to the resistance to antibiotics, resistance to disinfectant of *Salmonella* isolates was also investigated in this study (Supplementary Materials Table S2). The findings were as follows: as the QC strain, MIC values of *E. coli* ATCC 25922 to BC, BAB, and PMTS were determined to be 16 mg/L, 16 mg/L, and 2000 mg/L, respectively. The MIC values of the 190 isolates varied, with a range of 16–64 mg/L for BC and BAB, and from 2000 mg/L and 4000 mg/L for PMTS. The MIC value proportions of 190 isolates to three tested disinfectants are listed in Table 5. As for BAB, MIC of more than half of the isolates (61.05%, 116/190) was 64 mg/L. A MIC value of 2000 mg/L was obtained in 66.32% (126/190) of the isolates to PMTS; in addition, the MIC_50_ was 2000 mg/L, and the MIC_90_ was 4000 mg/L. Regarding BC, MIC of 47.89% (91/190) isolates was 32 mg/L, and MIC of 49.47% (94/190) isolates was 64 mg/mL; the MIC_50_ and MIC_90_ values were 32 mg/L and 64 mg/L, respectively.

Furthermore, the MIC_50_ and MIC_90_ values for the three disinfectants were generally consistent across isolates from different regions and types, with the exception of some differences in the MIC_50_ for the BC disinfectant. Specifically, the MIC_50_ for chicken-derived isolates from supermarkets A, C, and D was 64 mg/L for BC, while the MIC_50_ for pork-derived isolates was 32 mg/L for BC. Conversely, the MIC_50_ for pork-derived isolates from supermarket B was 64 mg/L for BC and 32 mg/L for chicken. The detailed information on the MIC values of *Salmonella* isolates from different regions and types is given in the Supplementary Materials Table S1.

Based on the MIC values obtained above, *Salmonella* isolates were considered “resistant to certain disinfectants” if they exhibited higher MIC values than those of the QC strain. Accordingly, 100% of the isolates in this study were found to be resistant to BAB, 97.37% were resistant to BC, and 33.6% were resistant to PMTS. In summary, the isolates in this study exhibited a higher resistance rate to BC and BAB, and a comparatively lower resistance rate to PMTS.

## 4. Discussion

In 2023, it was estimated that the incidence of *Salmonella* infections in China was 1295.59 per 100,000 population (95% uncertainty intervals: 1002.62, 1573.11), which was much higher than that in Europe in 2022 (15.3 per 100,000) and the United States in 2023 (13.9 per 100,000) [19,20,21]. Such higher contamination rates might result from the lack of specific standards on *Salmonella* spp. in fresh or frozen livestock/poultry products or during slaughtering and processing in China [19]. On the other side, the local wet markets were more common in China, and this might be an important source of *Salmonella* contamination.

In the present study, we collected 156 meat samples including pork and chicken sources from either supermarkets or a local wet market, aiming to first investigate the prevalence of *Salmonella*. Overall, 91 out of 156 (58.33%) samples were detected as *Salmonella*-positive. Notably, a lower overall contamination rate was found in the study of Tang et al. (7.95%) [22] and Aladi et al. (31.5%) [23]. In different countries and farms, variations in farming practices significantly influenced *Salmonella* prevalence and the inadequate coverage of cold chain logistics for agricultural products also posed challenge [24]. The centralization of meat supply in distinct production areas also played a key role in the high prevalence of *Salmonella*. Insufficient market regulation, possibly due to a suboptimal local retail environment, further exacerbated the issue [25,26]. In addition, the relatively lower prevalence of contamination in local wet markets as compared to the other two supermarkets could be attributed to the fact that local wet markets typically sell fresh meat. This meat was often processed on-site, with slaughtering, de-hairing, and evisceration completed within 20 min. As a result, the likelihood of cross-contamination was significantly reduced [27]. As for the contamination rate of different meat samples, a significant higher contamination rate in chicken (69.77%, 60/96) than pork (51.67%, 31/60) was revealed in our study. This was consistent with some previous reports: 42.3% for chicken and 8.9% for pork [28] and 22.2% for chicken and 6.7% for pork [29]. The prevalence of *Salmonella* was closely linked to environmental hygiene. The high rate of chicken contamination observed in this study may be attributed to the inadequate levels of disinfection in the abattoir or substandard hygiene practices.

In addition to the prevalence, identifying the serovars of *Salmonella* isolates was also an important step to figure out potential risk strains. Different serotypes of *Salmonella* exhibited specific host preferences and unique geographical distribution patterns. Serotyping enabled the tracing of *Salmonella* infection sources and provided insights into its transmission pathways and epidemiological trends. In 190 *Salmonella* isolates from 91 samples, nine serovars were identified, and the dominant serotype was *S.* London (46.84%, 89/190), followed by *S.* Typhimurium (10.00%, 19/190) and *S.* Enteritidis (8.42%, 16/190). Li et al. [30] analyzed sporadic diarrhea cases in China from 2014 to 2021, and found that *S.* London was one of the top five serotypes causing diarrhea cases. Meanwhile, *S.* London was one of the most common serotypes with an ACSuT (resistance to ampicillin, chloramphenicol, sulfonamide, and tetracycline) profile, which was similar to our study. The identification of serovars *S.* Typhimurium and *S.* Enteritidis in this study revealed potential food safety issue, since these two serovars were the primary *Salmonella* serovars involved in human infections globally over the years. Notably, the prevalence of *S.* Typhimurium (10.00%) and *S.* Enteritidis (8.42%) was higher than that found in Europe (4.00%, 7.90%) and America (2.00%, 2.00%) [20,31]. In EU and the US, their national control programs and specific standard restrictions on these two *Salmonella* serovars for meat production decreased the prevalence. Yet in China, no such measures were conducted, thus revealing their necessity in the future.

Antimicrobial resistance of pathogenic microorganisms was recognized as one of the most significant challenges to global public health. Numerous studies had focused on the antimicrobial resistance profiles of *Salmonella*, since its antimicrobial resistance could be transferred to the general population through *Salmonella*-contaminated meat. In the current study, all the isolated *Salmonella* strains were tested for antimicrobial susceptibility. The results showed that a high prevalence of antimicrobial resistance was observed among the *Salmonella* isolates, and 78.95% isolates were multi-drug-resistant, especially one *Salmonella* isolate was resistant to all the tested 10 antibiotics (Figure 1a). Therefore, these results indicated that the antimicrobial resistance situation of *Salmonella* was far from optimistic. Specifically, a high prevalence of resistance to ampicillin (83.16%), tetracycline (76.31%), and trimethoprim/sulfamethoxazole (67.37%) was identified in this study. Yang et al. [32] found that the *Salmonella* isolates from poultry were resistant to ampicillin (55.3%), tetracycline (47.8%), and trimethoprim/sulfamethoxazole (31.1%). This result reminded us that these antibiotics were commonly applied in animal breeding industry and may widely cause the resistance of *Salmonella*. On the other hand, *Salmonella* isolates from human diarrhea samples were resistant to ampicillin (73.4%), tetracycline (64.1%), and trimethoprim/sulfamethoxazole (34.9%), and the resistance rates increased by year from 2014 to 2021 [30]. Such similar antimicrobial resistance pattern of *Salmonella* isolated from meat and humans indicated the possibility of resistance transferring from meat to humans through the food chain. A low incidence of resistance to ampicillin/sulbactam (3.68%), ciprofloxacin (2.63%), and amikacin (0.53%) was found, which was consistent with previous research findings [32,33]. The low resistance to these antibiotics may be attributed to the fact that they were currently less utilized in animal husbandry or have only recently become widely available. Specifically, ciprofloxacin, a third-generation quinolone antimicrobial agent, was the primary treatment for *Salmonella* infections [34]. Fortunately, only five strains (2.63%) exhibited resistance to ciprofloxacin in the present study, and three of which were identified as *S.* Indiana. It has been observed that resistance to this class of antibiotics among *S.* Indiana is on the rise, with widespread resistant strains emerging [35].

Studies carried on disinfectant resistance could help optimizing disinfection strategies, and also enhance the understanding of the impact of disinfectants on antibiotic resistance, thereby providing strategies to mitigate antibiotic resistance. In this study, *Salmonella* isolates demonstrated resistance to the three disinfectants tested, and same results were revealed before [9,36]. Isolates obtained from the local wet market exhibited a lower rate of resistance to PMTS, yet displayed a similar rate of resistance to BC and BAB when compared to those from the three supermarkets. This result may be attributed to the fact that the source of meat in supermarkets was primarily from centralized abattoirs, which may involve disinfectant exposure, thereby leading to resistance to disinfectants. Resistance to both BC and BAB may be attributed to the widespread use of quaternary ammonium compounds disinfectants in recent years, which had facilitated the spread of certain resistance genes. This phenomenon warranted further investigation in the future. For different meat sources, isolates form pork exhibited higher resistance to PMTS compared to isolates from chicken, which may be a consequence of the more extensive use of PMTS in pig slaughterhouses.

## 5. Conclusions

In summary, *S.* London emerged as the predominant serovar among the *Salmonella* isolates obtained in this study. Meanwhile, the multi-drug-resistant *S.* Infantis was not detected in this study. This finding highlights that different serovars exhibit varying prevalence rates across Europe, the USA, and Asia. Additionally, the high levels of resistance to antibiotics and disinfectants observed in this study pose a potential risk to public health.

## Figures and Tables

**Table 1:** The information of the retail meat samples collected in this study.

Sample Source	Chicken Samples: Types and Quantities						Pork Samples: Types and Quantities			
	Chicken Wing Tips	Chicken Thighs	Chicken Wing Roots	Chicken Breasts	Chicken Wingettes	Chicken Necks	Pork Froth	**Sliced Pork**	**Pork Thighs**	**Shredded Pork**
Supermarket A	2	0	6	10	6	0	5	4	3	3
Supermarket B	6	4	4	6	4	0	3	8	3	1
Supermarket C	6	4	4	6	4	0	5	7	0	3
Local wet market	4	6	0	6	6	2	5	7	0	3
Total	18	14	14	28	20	2	18	26	6	10

**Table 2:** Serovars information of the *Salmonella* isolates.

Serogroups	Serotypes	Total	Sample Source				Sample Type	
			A	B	C	D	Pork	Chicken
Group O:4 (B)	*S.* Typhimurium	19	4	1	10	4	9	10
	*S.* Indiana	3	3	0	0	0	0	3
	*S*. Essen	1	1	0	0	0	0	1
	-	20	4	3	4	9	9	11
Group O:7 (C1)	-	15	0	1	7	7	12	3
Group O:8 (C_2_-C_3_)	*S.* Gold Coast	8	5	1	0	2	8	0
	*S*. Kentucky	1	1	0	0	0	0	1
Group O:9 (D_1_)	*S*. Enteritidis	16	11	4	1	0	0	16
	-	4	4	0	0	0	0	4
Group O:3,10 (E_1_)	*S*. London	89	22	31	28	8	18	71
	*S.* Muenster	13	0	0	3	10	0	13
Group O:1,3,19 (E_4_)	*S.* Hayindogo	1	0	0	0	1	0	1

Note: “-” represented un-typeable serotypes.

**Table 3:** The antimicrobial susceptibility results at the primary serovar level.

Serovars	Antibiotics										**MDR**			
	SXT	TET	CIP	AK	CZ	GEN	AAM	NN	AMP	**AMC**	**≥1**	**≥3**	**≥5**	**≥7**
*S.* London (*n* = 89)	75	69	0	0	0	27	0	0	75	43	75	75	14	0
*S.* Typhimurium (*n* = 19)	12	18	0	0	10	3	3	9	18	18	18	18	11	3
*S.* Enteritidis (*n* = 16)	2	4	0	0	0	0	1	0	10	9	10	7	0	0
*S.* Muenster (*n* = 13)	13	13	0	0	13	13	0	1	13	10	13	13	13	1
*S.* Gold Coast (*n* = 8)	2	2	0	0	6	0	0	0	7	2	8	3	0	0

Note: SXT, trimethoprim/sulfamethoxazole; TET, tetracycline; CIP, ciprofloxacin; AK, amikacin; CZ, cefazolin; GEN, gentamycin; AAM, ampicillin/sulbactam; NN, tobramycin; AMC, amoxicillin/clavulanic acid.

**Table 4:** The antimicrobial resistance profile of *Salmonella* isolates.

Antimicrobial Resistance Profile	Sample Source				Sample Type		Total
	A	B	C	D	Pork	Chicken	
TET			3		3		3
AMP		1				1	1
CZ-AMP	3	1			4		4
TET-NN		1		2		3	3
SXT-TET			2		2		2
CZ-AMC	1				1		1
AMP-AMC	3			1	1	3	4
SXT-TET-AMP	5	7	9	4	5	20	25
TET-AMP-AMC	5	2	7		8	6	14
SXT-AMP-AMC	4		1		1	4	5
TET-NN-AMC			1		1		1
TET-NN-AMP	1				1		1
SXT-GEN-AMP	1	1		1	1	2	3
AAM-AMP-AMC	1					1	1
CZ-AMP-AMC	1				1		1
SXT-TET-GEN-AMP	2	2	2	1	2	5	7
SXT-TET-AMP-AMC	8	6	9	11	11	23	34
SXT-GEN-AMP-AMC		1	2		1	2	3
TET-CIP-NN-AMP	1					1	1
TET-NN-AMP-AMC	1			1	2		2
SXT-TET-GEN-AMP-AMC	1	7	6		1	13	14
SXT-TET-CZ-AMP-AMC			1	6	1	6	7
TET-CIP-NN-AMP-AMC	2					2	2
SXT-TET-CZ-GEN-AMP			2	1		3	3
SXT-TET-NN-AMP-AMC			1	1	2		2
SXT-TET-CZ-NN-AMP-AMC	3	1	1		1	4	5
SXT-TET-CZ-GEN-AMP-AMC			1	9		10	10
SXT-TET-CZ-GEN-NN-AMP-AMC				1		1	1
SXT-TET-CIP-CZ-GEN-AMP-AMC	1					1	1
SXT-TET-CZ-AAM-NN-AMP-AMC			2			2	2
SXT-TET-CZ-GEN-NN-AMP-AMC				1		1	1
SXT-TET-CZ-GEN-AAM-NN-AMP-AMC			2	1		3	3
SXT-TET-CIP-AK-CZ-GEN-AAM-NN-AMP-AMC	1				1		1

Note: SXT, trimethoprim/sulfamethoxazole; TET, tetracycline; CIP, ciprofloxacin; AK, amikacin; CZ, cefazolin; GEN, gentamycin; AAM, ampicillin/sulbactam; NN, tobramycin; AMC, amoxicillin/clavulanic acid.

**Table 5:** The MIC value proportions of 190 isolates to three tested disinfectants.

Disinfectants	MIC (mg/L)				
	16	32	64	2000	4000
BC	2.63%	47.89%	49.47%		
BAB	0.53%	38.42%	61.05%		
PMTS				66.32%	33.68%

Note: BC, benzalkonium chloride; BAB, benzalkonium bromide; PMTS, potassium monopersulfate triple salt.

## Data Availability

The original contributions presented in the study are included in the article/Supplementary Materials, and further inquiries can be directed to the corresponding authors.

## Supplementary Materials

The following supporting information can be downloaded at: https://www.mdpi.com/article/10.3390/pathogens14030222/s1, Table S1: The MIC values of *Salmonella* isolates from different regions and types; Table S2: Antibiotic and disinfectant resistance in 190 isolates.

## Abbreviations

The following abbreviations are used in this manuscript:

BC	Benzalkonium chloride
BAB	Benzalkonium bromide
PMTS	Potassium monopersulfate triple salt
MIC	Minimum inhibitory concentration
MIC_50_	50% minimum inhibitory concentration
MIC_90_	90% minimum inhibitory concentration
NTS	Non-typhoidal *Salmonella*
MDR	Multi-drug-resistant
TTB	Tetrathionate broth
SC	Selenite cysteine
XLD	Xylose lysine desoxycholate
AMP	Ampicillin
CZ	Cefazolin
AAM	Ampicillin/sulbactam
AMC	Amoxicillin/clavulanic acid
CIP	Ciprofloxacin
AK	Amikacin
GEN	Gentamycin
NN	Tobramycin
TET	Tetracycline
SXT	Trimethoprim/sulfamethoxazole
MHB	Mueller–Hinton broth
QC	Quality control
S	Susceptible
I	Intermediate
R	Resistance
