# Supplementary material for: Prevalence and Antimicrobial Susceptibility of Salmonella in Retail Meat Collected from Different Markets in Sichuan, China
Source: Pathogens. 2025 Feb 25;14(3):222. doi: 10.3390/pathogens14030222 (PMC11944474; doi:10.3390/pathogens14030222)
Supplement: Supplementary file 1 [file pathogens-14-00222-s001.zip › pathogens-3484292-supplementary.pdf]

Supplementary Materials

Prevalence and Antimicrobial Susceptibility of *Salmonella* in Retail Meat Collected from Different Markets in Sichuan, China

Hang Zeng [1,2,*,†], Donghai Yang [1,†], Nanxi Huang [1], Yonglin Li [1], Jiazhen Chen [1], Zhongjia Yu [3], Jie Tang [1,2], Zhenju Jiang [1,*]

[1] School of Food and Bioengineering, Xihua University, Chengdu 610039, China; dhyang0701@foxmail.com (D.Y.); hnxhhh@163.com (N.H.); lyl15283671501@foxmail.com (Y.L.); chenjiazhencjzcjz@163.com (J.C.); tangjie1225@mail.xhu.edu.cn (J.T.)

[2] Food Microbiology Key Laboratory of Sichuan Province, Xihua University, Chengdu 610039, China

[3] School of Animal Science and Technology, Foshan University, Foshan 528225, China; yuzhongjia@fosu.edu.cn

* Correspondence: zenghang@mail.xhu.edu.cn (H.Z.); zhjjiang@mail.xhu.edu.cn (Z.J.)

† These authors contributed equally to this work.

Supplementary Materials

Table S1. The MIC values of *Salmonella* isolates from different regions and types.

Region of samples	Type of samples	Total	Disinfectant MIC		
			BAB (mg/mL)	BC (mg/mL)	PMTS (g/L)
A	Chicken	1	16		
		21	32		
		24	64		
		28			2
		18			4
		3		16	
		17		32	
		26		64	
	Pork	4	32		
		5	64		
		7			2
		2			4
		7		32	
		2		64	
B	Chicken	10	32		
		20	64		
		21			2
		9			4
		17		32	
		13		64	
	Pork	3	32		
		7	64		
		6			2
		4			4

Supplementary Materials

		3		32	
		7		64	
		14	32		
		15	64		
	Chicken	15			2
		14			4
		12		32	
C		17		64	
		6	32		
		18	64		
	Pork	19			2
		5			4
		15		32	
		9		64	
		10	32		
		18	64		
		18			2
	Chicken	10			4
		2		16	
		14		32	
D		12		64	
		5	32		
		9	64		
	Pork	12			2
		2			4
		6		32	
		8		64	

Note: BAB=benzalkonium bromide, BC=benzalkonium chloride, PMTS=Potassium monopersulfate triple salt.

Table S2. Antibiotic and disinfectant resistance in 190 isolates.

NO.	Region	Type	Antibiotic resistance										Disinfectant MIC		
			SXT	TET	CIP	AK	CZ	GEN	AAM	NN	AMP	AMC	BAB (mg/mL)	BC (mg/mL)	PMTS (g/L)
SC-42	A	Chicken	R	R	I	S	S	S	S	S	R	R	32	16	4
BS-TTB-68	B	Chicken	R	R	I	S	S	R	S	I	R	R	32	64	4
SC-87	C	Chicken	R	R	I	S	I	R	S	I	R	R	32	32	4
TTB-42	A	Chicken	S	S	S	S	S	S	S	S	I	S	32	32	4
TTB-40	B	Chicken	R	R	I	S	S	S	S	S	R	I	32	32	4
BS-TTB-91	D	Chicken	S	R	I	S	S	I	S	R	S	S	32	32	4
BS-TTB-61	D	Chicken	R	R	S	S	R	R	S	S	R	R	32	16	4
TTB-Z53	A	Pork	S	S	S	S	R	S	S	S	R	I	32	32	4
BS-TTB-42	A	Chicken	R	R	I	S	S	S	S	S	R	I	32	32	2
SC-46	A	Chicken	R	S	S	S	I	S	S	S	R	R	32	16	4
TTB-39	B	Chicken	R	R	S	S	S	S	S	S	R	I	32	32	4
TTB-52	C	Chicken	R	R	I	S	R	R	R	R	R	R	64	32	4
SC-86	C	Chicken	R	R	I	S	I	S	S	S	R	R	32	32	4
SC-43	A	Chicken	S	R	S	S	I	S	S	S	R	R	32	64	2
BS-SC-Z14	C	Pork	S	R	S	S	I	S	S	S	R	R	32	32	4
BS-TTB-96	D	Chicken	R	R	S	S	R	S	S	S	R	R	32	64	4
BS-TTB-95	D	Chicken	R	R	I	S	R	S	S	S	R	R	32	32	4
TTB-80	A	Chicken	R	R	I	S	S	S	S	S	R	R	16	32	4
BS-TTB-41	A	Chicken	R	R	I	S	S	S	S	S	R	I	32	64	2
BS-TTB-Z40	D	Pork	S	S	S	S	S	S	S	S	S	S	64	32	2
TTB-57	D	Chicken	R	R	I	S	S	S	S	S	R	I	64	32	2
BS-TTB-Z17	C	Pork	S	R	S	S	S	S	S	S	S	S	64	32	2
BS-TTB-Z26	D	Pork	R	R	I	S	S	S	S	S	R	I	64	32	2

Supplementary Materials

BS-TTB-Z36	A	Pork	S	S	I	S	R	S	S	S	R	I	64	32	2
BS-TTB-70	B	Chicken	R	R	I	S	S	I	S	S	R	I	64	32	2
TTB-Z16	C	Pork	R	R	S	S	S	S	S	S	S	S	64	32	2
SC-77	A	Chicken	R	S	I	S	I	S	S	S	R	R	64	32	2
SC-88	C	Chicken	R	R	I	S	I	S	S	S	R	I	64	32	2
TTB-33	B	Chicken	S	S	I	S	S	S	S	S	S	S	64	32	2
SC-37	B	Chicken	R	R	I	S	I	S	S	S	R	R	64	32	2
BS-TTB-Z27	D	Pork	R	R	I	S	S	S	S	S	R	R	64	32	2
BS-TTB-54	C	Chicken	R	R	I	S	R	R	S	S	R	I	64	32	2
BS-TTB-60	D	Chicken	R	R	I	S	R	R	I	S	R	R	64	64	2
BS-SC-70	B	Chicken	R	R	I	S	I	R	S	I	R	R	64	32	2
TTB-48	A	Chicken	R	R	I	S	I	S	S	S	R	R	64	32	2
TTB-Z52	A	Pork	S	S	S	S	R	S	S	S	S	R	64	32	2
SC-11-2	A	Chicken	S	R	R	S	S	I	S	R	R	R	64	32	2
BS-TTB-62	D	Chicken	R	R	I	S	R	R	I	R	R	R	64	32	2
SC-78	A	Chicken	S	R	I	S	I	S	S	S	R	R	64	32	2
TTB-51	C	Chicken	R	R	I	S	R	R	S	S	R	I	64	64	4
TTB-54	C	Chicken	R	R	I	S	R	R	S	S	R	R	64	64	2
TTB-35	B	Chicken	R	R	I	S	S	S	S	S	R	I	64	32	2
TTB-Z2B	C	Pork	R	R	I	S	S	S	S	S	R	I	64	32	2
TTB-60	D	Chicken	R	R	I	S	R	R	S	S	R	R	64	32	2
SC-33	B	Chicken	S	S	I	S	S	S	S	S	S	S	64	32	2
TTB-93	D	Chicken	R	R	I	S	R	S	S	S	R	R	64	16	2
BS-TTB-11	A	Chicken	I	R	R	S	S	I	S	R	R	R	64	16	2
TTB-61A	D	Chicken	R	R	I	S	R	R	S	S	R	R	64	32	2
BS-TTB-Z56	B	Pork	S	R	I	S	S	S	S	S	R	R	64	32	2
SC-40	B	Chicken	R	R	I	S	I	S	S	S	R	I	64	32	2
BS-TTB-72	B	Chicken	R	I	I	S	S	R	S	I	R	R	64	32	2

BS-TTB-69	B	Chicken	R	R	I	S	S	S	S	S	R	R	64	32	2
TTB-83	C	Chicken	R	R	S	S	S	R	S	S	R	R	64	32	2
TTB-37	B	Chicken	R	R	I	S	S	S	S	S	R	R	64	32	2
BS-TTB-Z14	C	Pork	S	R	S	S	S	S	S	S	R	R	64	32	2
BS-TTB-77A	A	Chicken	R	R	I	S	S	S	S	S	R	R	64	32	2
BS-TTB-83	C	Chicken	R	R	I	S	I	R	S	S	R	R	64	32	2
TTB-91	D	Chicken	S	R	I	S	S	I	S	R	S	S	64	32	2
BS-TTB-74	A	Chicken	R	R	I	S	S	S	S	S	R	R	64	32	2
BS-TTB-Z43	C	Pork	R	R	I	S	S	S	S	S	R	R	64	32	2
BS-TTB-Z3	C	Pork	S	R	S	S	S	I	S	R	I	R	64	32	2
TTB-61B	D	Chicken	R	R	I	S	R	R	S	I	R	R	64	32	2
TTB-Z50	A	Pork	S	R	I	S	I	S	S	S	R	R	32	32	2
BS-TTB-Z13	C	Pork	R	S	I	S	I	S	S	S	R	R	32	32	2
TTB-58	D	Chicken	R	R	I	S	S	S	S	S	R	R	64	32	2
BS-TTB-76	A	Chicken	R	R	I	S	R	I	I	R	R	R	64	64	2
TTB-62A	D	Chicken	R	R	I	S	R	R	S	S	R	I	64	32	2
TTB-62B	D	Chicken	R	R	I	S	R	R	S	I	R	R	64	64	2
BS-TTB-Z37	D	Pork	R	R	I	S	I	I	I	R	R	R	64	64	2
TTB-11-1B	A	Chicken	S	R	R	S	S	I	S	R	R	I	64	64	4
SC-39	B	Chicken	S	S	S	S	S	S	S	S	S	S	64	64	4
TTB-81	C	Chicken	R	R	I	S	S	I	S	S	R	I	64	64	4
TTB-63	D	Chicken	R	R	I	S	R	R	S	S	R	R	64	64	2
TTB-46	A	Chicken	R	S	I	S	I	S	S	S	R	R	64	32	4
TTB-77	A	Chicken	R	R	I	S	I	R	S	S	R	I	64	64	4
BS-TTB-48	A	Chicken	S	S	I	S	S	S	S	S	S	S	64	32	2
SC-52	C	Chicken	R	R	I	S	S	I	S	S	R	R	64	64	4
BS-TTB-Z53	A	Pork	S	S	S	S	R	S	S	S	R	I	64	32	4
BS-TTB-Z16	C	Pork	R	R	S	S	S	S	S	S	S	S	64	64	4

Supplementary Materials

TTB-Z54	A	Pork	S	R	S	S	S	S	S	R	R	I	64	64	2
BS-TTB-Z38	D	Pork	R	R	I	S	S	S	S	S	R	R	64	64	2
TTB-67	B	Chicken	R	R	I	S	S	R	S	S	R	R	64	32	2
TTB-43	A	Chicken	S	S	S	S	S	S	S	S	S	S	64	32	4
TTB-41	A	Chicken	R	R	I	S	S	S	S	S	R	I	64	32	2
TTB-76	A	Chicken	R	R	R	R	R	R	R	R	R	R	64	32	4
TTB-Z26	D	Pork	R	R	I	S	S	S	S	S	R	I	32	32	2
BS-TTB-Z57	B	Pork	S	S	S	S	R	S	S	S	R	I	32	32	4
TTB-72	B	Chicken	S	R	S	S	I	S	I	S	R	R	32	32	2
BS-TTB-Z18	C	Pork	S	R	I	S	I	S	S	S	S	S	32	32	4
TTB-Z1	C	Pork	R	R	I	S	S	I	S	R	R	R	64	32	2
BS-TTB-67	B	Chicken	R	R	I	S	S	R	S	I	R	R	64	32	4
SC-85	C	Chicken	R	R	I	S	S	S	S	S	R	S	64	32	2
BS-TTB-Z15	C	Pork	R	I	I	S	S	R	S	S	R	R	32	32	4
BS-TTB-66	B	Chicken	R	R	I	S	S	S	S	S	R	S	64	32	2
TTB-95	D	Chicken	R	R	I	S	S	I	S	S	R	R	64	64	2
TTB-64A	D	Chicken	R	R	I	S	R	R	S	S	R	R	64	32	2
BS-SC-71	B	Chicken	S	S	S	S	S	S	S	S	S	S	64	64	2
BS-TTB-Z46	C	Pork	R	R	I	S	S	S	S	S	R	R	64	64	2
BS-TTB-Z55	B	Pork	S	S	S	S	S	S	S	S	S	S	64	64	2
BS-TTB-Z54	A	Pork	S	R	S	S	S	I	I	R	R	R	64	64	2
TTB-51-2	C	Chicken	R	R	I	S	R	R	R	R	R	R	32	64	2
BS-TTB-Z7	B	Pork	R	R	I	S	S	R	S	I	R	R	64	64	4
BS-SC-10	A	Chicken	S	S	S	S	S	S	S	S	S	S	64	64	2
BS-SC-Z7	B	Pork	S	S	S	S	S	S	S	S	S	S	64	64	4
TTB-Z7	B	Pork	R	I	I	S	S	R	S	S	R	I	64	64	2
TTB-Z55	B	Pork	S	S	S	S	S	S	S	S	S	S	64	64	4
BS-TTB-81	C	Chicken	R	R	I	S	S	R	S	S	R	I	64	64	4

BS-TTB-65	B	Chicken	S	R	I	S	S	S	S	R	S	S	32	64	4
TTB-85	C	Chicken	R	R	I	S	R	I	I	R	R	R	32	32	2
TTB-66	B	Chicken	R	R	I	S	S	S	S	S	R	I	32	64	2
TTB-Z40	D	Pork	R	R	I	S	S	R	S	I	R	I	32	64	2
TTB-Z46	C	Pork	R	R	I	S	I	S	S	S	R	R	64	64	2
TTB-69	B	Chicken	R	R	I	S	S	R	S	I	R	I	32	64	2
SC-69	B	Chicken	R	R	I	S	R	I	I	R	R	R	32	64	2
SC-Z58B	B	Pork	S	S	S	S	S	S	S	S	S	S	32	64	2
TTB-78	A	Chicken	R	I	I	S	S	R	S	S	R	I	32	64	2
TTB-86	C	Chicken	R	R	S	S	R	S	R	R	R	R	32	64	2
TTB-Z43	C	Pork	R	R	I	S	I	S	S	S	R	R	64	64	4
BS-TTB-Z41	D	Pork	R	R	S	S	I	S	S	S	R	R	32	64	2
TTB-84	C	Chicken	R	R	I	S	S	S	S	S	R	I	32	64	2
TTB-73	A	Chicken	S	R	S	S	I	S	I	S	R	R	32	64	2
TTB-Z41	D	Pork	R	R	S	S	S	S	S	S	R	I	32	64	2
TTB-74	A	Chicken	R	R	I	S	S	S	S	S	R	I	64	64	2
TTB-88	C	Chicken	R	R	I	S	S	R	S	S	R	I	32	64	2
BS-TTB-75	A	Chicken	R	R	I	S	S	R	S	S	R	I	32	64	4
TTB-94	D	Chicken	R	I	I	S	S	R	S	I	R	I	32	64	4
TTB-68	B	Chicken	R	R	I	S	S	R	S	I	R	R	32	64	4
BS-TTB-93	D	Chicken	R	R	S	S	R	S	S	S	R	R	32	64	4
SC-82	C	Chicken	R	R	I	S	S	S	S	S	R	I	32	32	4
SC-81	C	Chicken	R	R	I	S	S	R	S	S	R	R	32	64	4
BS-TTB-87	C	Chicken	R	R	I	S	I	R	S	S	R	R	32	64	4
BS-SC-78	A	Chicken	R	R	R	S	R	R	S	I	R	R	32	64	4
BS-TTB-89	D	Chicken	S	S	S	S	S	S	S	S	S	S	32	64	4
BS-TTB-94	D	Chicken	R	R	I	S	R	S	S	S	R	R	32	32	4
BS-TTB-86A	C	Chicken	R	I	I	S	S	R	S	S	R	R	32	32	4

Supplementary Materials

BS-TTB-85	C	Chicken	R	R	I	S	S	R	S	S	R	R	32	64	4
BS-TTB-78	A	Chicken	R	R	I	S	I	R	S	S	R	R	32	64	4
BS-TTB-77B	A	Chicken	R	R	I	S	I	I	S	I	R	R	32	64	4
TTB-75	A	Chicken	S	S	S	S	S	S	S	S	S	S	32	64	4
TTB-65	B	Chicken	R	R	I	S	S	I	S	S	R	R	64	64	2
BS-TTB-80	A	Chicken	S	S	S	S	S	S	S	S	S	S	32	64	2
BS-TTB-84	C	Chicken	R	R	I	S	I	I	S	S	R	I	64	64	2
BS-SC-81	C	Chicken	R	R	I	S	S	I	S	S	R	R	32	64	2
TTB-96	D	Chicken	R	R	S	S	R	S	S	S	R	R	32	64	2
BS-TTB-Z2	C	Pork	R	R	I	S	S	S	S	S	R	I	32	64	2
TTB-Z2A	C	Pork	R	R	I	S	S	S	S	S	R	R	64	64	2
TTB-15-1	A	Chicken	S	S	S	S	S	S	S	S	S	S	32	64	2
BS-TTB-Z12	D	Pork	S	S	S	S	S	S	S	S	R	R	64	64	2
TTB-10	A	Chicken	S	S	I	S	S	S	S	S	S	S	32	64	2
SC-15-2	A	Chicken	S	S	S	S	S	S	S	S	R	R	32	64	2
SC-10	A	Chicken	S	S	S	S	S	S	R	S	R	R	32	64	2
SC-9A	A	Chicken	R	S	S	S	S	S	S	S	R	R	32	32	2
TTB-Z1-2	C	Pork	R	R	I	S	S	S	S	S	R	R	32	64	2
BS-SC-Z7-2	B	Pork	S	S	S	S	S	S	S	S	S	S	32	64	2
SC-6-2	B	Chicken	S	S	S	S	I	S	S	S	R	I	64	64	4
SC-Z7	B	Pork	R	R	I	S	I	R	S	I	R	I	64	32	2
TTB-3	B	Chicken	S	R	S	S	S	R	S	R	R	R	32	32	4
TTB-9	A	Chicken	S	R	S	S	I	S	S	S	R	R	32	64	2
BS-TTB-49	C	Chicken	R	R	I	S	S	S	S	S	R	I	32	64	4
BS-SC-86	C	Chicken	R	R	I	S	R	I	R	R	R	R	64	64	2
TTB-16	A	Chicken	S	S	I	S	I	S	S	S	R	R	64	64	4
BS-TTB-Z6	A	Pork	R	R	I	S	R	I	I	R	R	R	32	32	2
TTB-18	C	Chicken	S	R	S	S	S	S	S	S	R	R	64	64	4

Supplementary Materials

TTB-Z12	D	Pork	R	R	I	S	S	S	S	S	R	R	32	32	2
SC-9B	A	Chicken	S	S	S	S	I	S	S	S	R	R	64	64	4
BS-SC-43	A	Chicken	R	R	I	S	R	I	I	R	R	R	32	32	2
BS-TTB-43	A	Chicken	S	S	S	S	S	S	S	S	S	S	64	64	4
BS-SC-42A	A	Chicken	R	R	S	S	S	S	S	S	R	R	64	64	2
BS-SC-42B	A	Chicken	R	R	I	S	S	S	S	S	R	I	64	64	4
BS-TTB-58	D	Chicken	R	R	S	S	S	S	S	S	R	R	64	64	4
BS-TTB-57	D	Chicken	R	R	S	S	S	S	S	S	R	R	64	64	4
TTB-Z25	D	Pork	R	R	S	S	S	S	S	S	R	R	64	64	2
TTB-Z18	C	Pork	S	R	S	S	S	S	S	S	S	S	64	64	2
TTB-Z17	C	Pork	S	R	S	S	S	S	S	S	R	R	64	32	2
TTB-Z15	C	Pork	R	R	S	S	R	S	S	S	R	R	64	64	2
SC-Z14	C	Pork	S	R	S	S	S	S	S	S	R	R	64	32	2
TTB-Z14	C	Pork	S	R	S	S	S	S	S	S	R	R	64	32	2
TTB-Z13	C	Pork	S	R	S	S	S	S	I	S	R	R	64	32	2
BS-TTB-44	A	Chicken	R	R	S	S	S	S	S	S	R	R	64	32	2
BS-SC-44A	A	Chicken	S	S	S	S	S	S	S	S	S	S	64	64	2
TTB-64B	D	Chicken	R	R	I	S	R	R	S	S	R	R	64	32	2
BS-TTB-31	D	Chicken	R	R	I	S	R	R	R	R	R	R	32	32	2
BS-TTB-63	D	Chicken	R	R	I	S	S	S	S	S	R	R	64	64	2
BS-TTB-39A	B	Chicken	R	R	I	S	S	S	S	S	R	R	64	64	2
BS-SC-37	B	Chicken	S	S	S	S	S	S	S	S	S	S	64	64	2
BS-TTB-35	B	Chicken	R	R	I	S	S	R	S	S	R	R	64	64	2
BS-SC-49	C	Chicken	R	R	S	S	S	S	S	S	R	R	64	32	2
TTB-Z36	A	Pork	S	S	S	S	R	S	S	S	R	R	32	32	2
SC-Z30	D	Pork	S	R	I	S	S	I	I	R	R	R	64	64	4
TTB-Z27	D	Pork	R	R	S	S	S	S	S	S	R	R	64	32	4

Supplementary Materials

Note: SXT=Trimethoprim/Sulfamethoxazole, TET=Tetracycline, CIP=Ciprofloxacin, AK=Amikacin, CZ=Cefazolin, GEN=Gentamycin, AAM=Ampicillin/Sulbactam, NN=Tobramycin, AMC=Amoxicillin/Clavulanic acid; BAB=benzalkonium bromide, BC=benzalkonium chloride, PMTS=Potassium monopersulfate triple salt; R=resistance, I=intermediate, S=susceptible.
